# Supplementary material for: Acoustic delivery of indocyanine green via biosynthetic gas vesicles for tumor photothermal therapy
Source: PLoS Biol. 2026 May 13;24(5):e3003786. doi: 10.1371/journal.pbio.3003786 (PMC13170872; doi:10.1371/journal.pbio.3003786)
Supplement: S1 Materials and Methods — (DOCX) [file pbio.3003786.s002.docx]

**Visibly acoustic delivery of ICG via biosynthetic gas vesicles for tumor photothermal therapy**

**Materials and Methods**

**Pharmacokinetic analysis**

To evaluate the circulation behavior of ICG-GVs, healthy female C57BL/6 mice (6–8 weeks old, 15–20 g) were randomly divided into two groups (n=3 per group). Mice were intravenously injected via the tail vein with either free ICG or ICG-GVs at an equivalent ICG dose of 2 mg/kg. Blood samples were collected from the orbital sinus at predetermined time points: 1, 5, 10, 30 minutes and 1, 2, 4, 8, 12, 24 hours post-injection post-injection. Blood samples were immediately centrifuged at 3000 rpm for 10 min at 4°C to obtain plasma. The concentration of ICG in plasma was measured using a fluorescence spectrophotometer (FluoroMax-4, Horiba, Japan) with excitation at 780 nm and emission at 820 nm.
