# Supplementary figures and images for: Acoustic delivery of indocyanine green via biosynthetic gas vesicles for tumor photothermal therapy
Source: PLoS Biol. 2026 May 13;24(5):e3003786. doi: 10.1371/journal.pbio.3003786 (PMC13170872; doi:10.1371/journal.pbio.3003786)

A

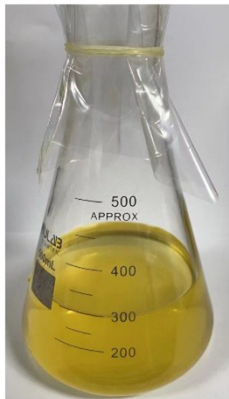

B

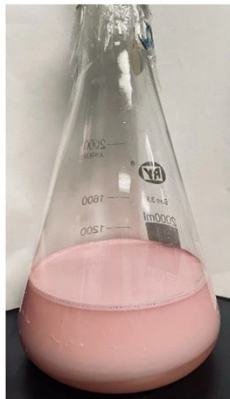

C

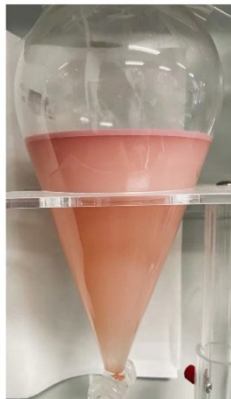

D

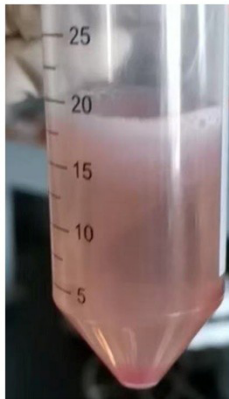

E

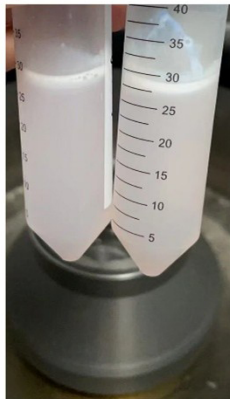

F

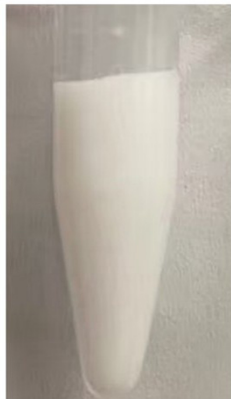

Supplement: S1 Fig — Schematic illustration of the procedure for isolation and purification of GVs from Halobacterium NRC-1. (A) Initial culture system. (B) Mature bacterial liquid system after cultivation. (C) Bacterial liquid transferred into a separatory funnel awaiting collection. (D) GVs obtained after the first centrifugation. (E) GVs obtained after the final centrifugation. (F) GVs collected together. (PDF) [file pbio.3003786.s003.pdf]

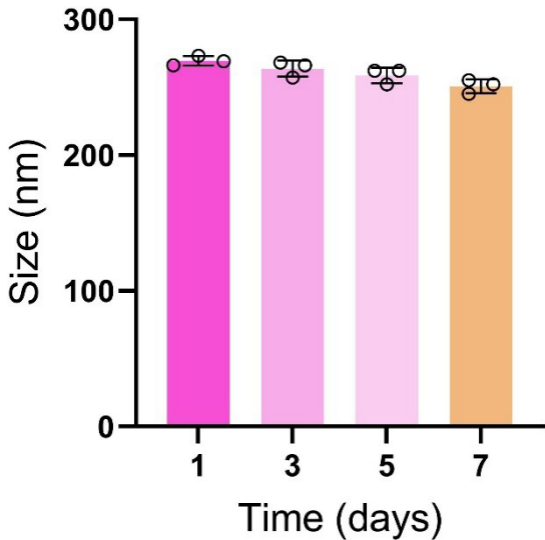

Supplement: S2 Fig — The stability of ICG-GVs was monitored in PBS over 7 days. Data are presented as mean ± SD. Each dot represents an individual measurement (n = 3 per group). The underlying numerical data for this figure can be found in S1 Data. (PDF) [file pbio.3003786.s004.pdf]

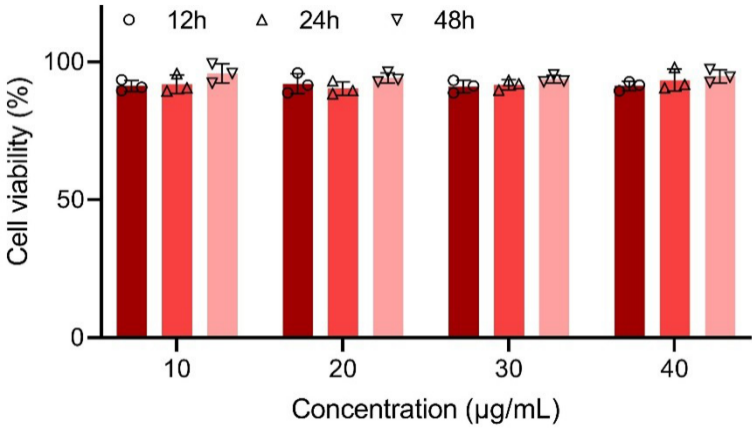

Supplement: S3 Fig — Assessment of MB49 cell viability following a 48-hour exposure to ICG-GVs (10–40 μg/mL). Data are presented as mean ± SD. Each dot represents an individual measurement (n = 3 per group). The underlying numerical data for this figure can be found in S1 Data. (PDF) [file pbio.3003786.s005.pdf]

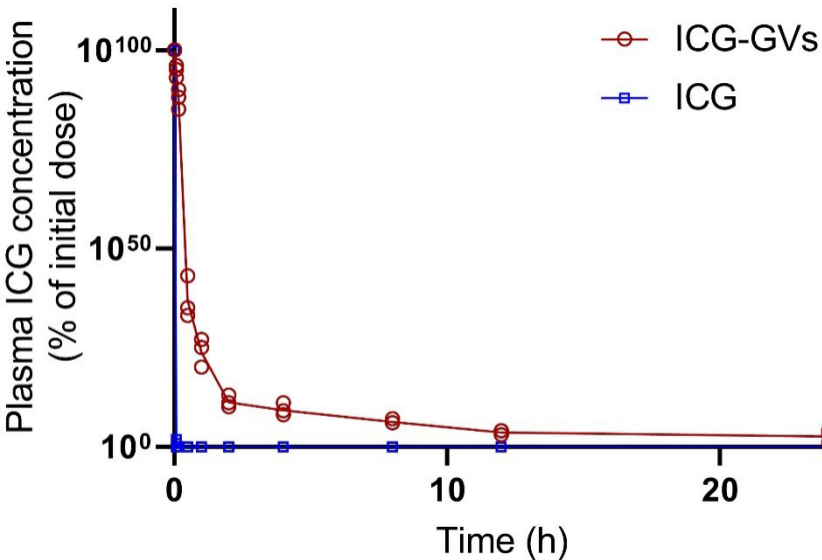

Supplement: S4 Fig — Plasma concentration-time profiles of ICG after intravenous injection of free ICG or ICG-GVs (ICG dose: 2 mg/kg) in healthy C57BL/6 mice. Blood samples were collected at indicated time points and ICG concentration was measured by fluorescence spectrophotometry. Data are presented as mean ± SD. Each dot represents an individual measurement (n = 3 per group). The underlying numerical data for this figure can be found in S1 Data. (PDF) [file pbio.3003786.s006.pdf]

A

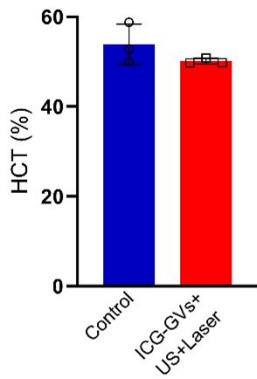

B

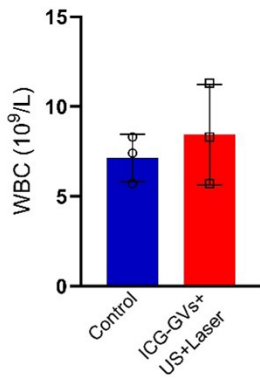

C

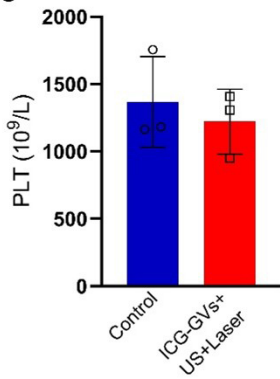

D

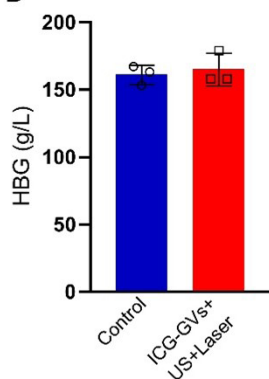

E

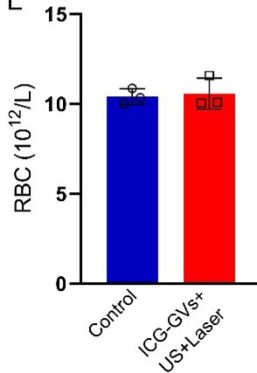

F

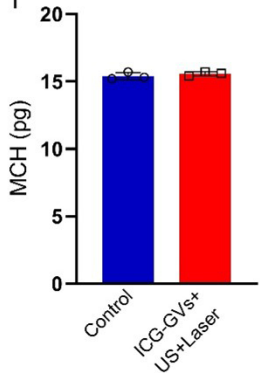

G

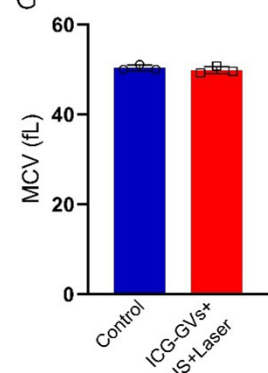

H

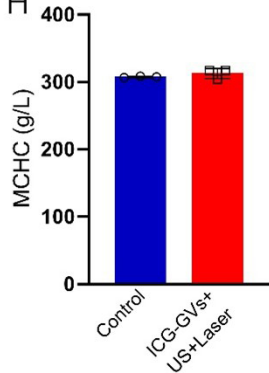

Supplement: S5 Fig — Hematology profiles, (A) Hematocrit (HCT), (B) white blood cell count (WBC), (C) platelet count (PLT), (D) hemoglobin (HGB), (E) red blood cell count (RBC), (F) mean corpuscular hemoglobin (MCH), (G) mean corpuscular volume (MCV), and (H) mean corpuscular hemoglobin concentration (MCHC), show no significant differences between the control and the ICG-GVs + US + Laser treatment groups. Data are presented as mean ± SD. Each dot represents an individual measurement (n = 3 per group). The underlying numerical data for this figure can be found in S1 Data. (PDF) [file pbio.3003786.s007.pdf]

A

ALT (U/L)

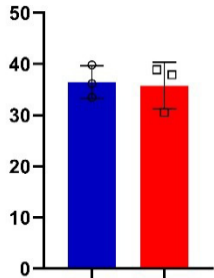

B

AST (U/L)

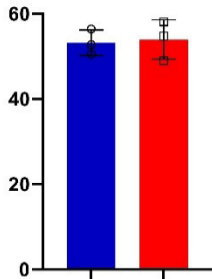

C

BUN (mg/dL)

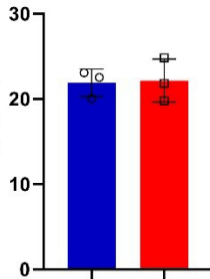

D

CREA ( $\mu$ mol/L)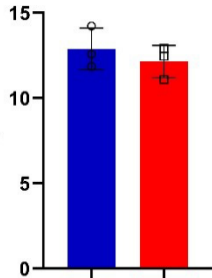

Supplement: S6 Fig — (A) ALT, (B) AST, (C) BUN, and (D) CREA levels in control and ICG-GVs + US+Laser treated groups. Data are presented as mean ± SD. Each dot represents an individual measurement (n = 3 per group). The underlying numerical data for this figure can be found in S1 Data. (PDF) [file pbio.3003786.s008.pdf]

Heart

Liver

Spleen

Lung

Kidney

Control

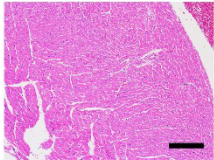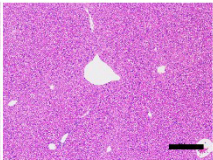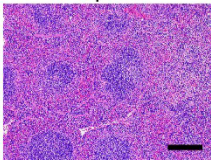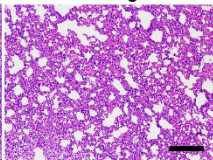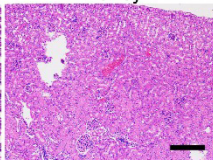ICG-GVs+  
US+Laser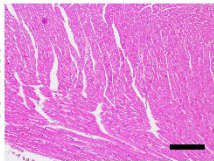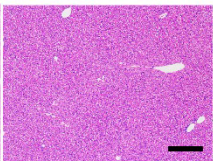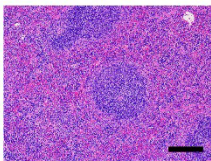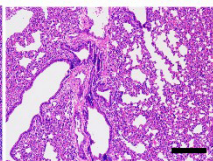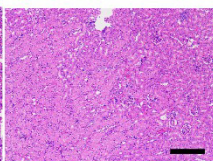

Supplement: S7 Fig — H&E staining of major organs (heart, liver, spleen, lung, kidney) in control and ICG-GVs group. Scale bar = 200 μm. (PDF) [file pbio.3003786.s009.pdf]

Control

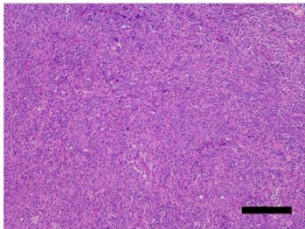

US+Laser

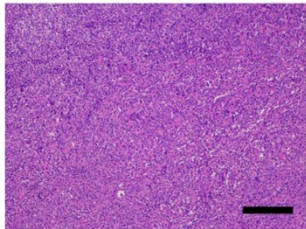

ICG-GVs

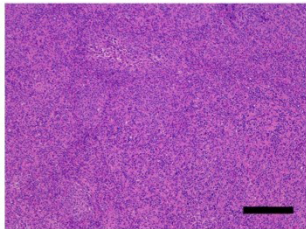

IGV+US

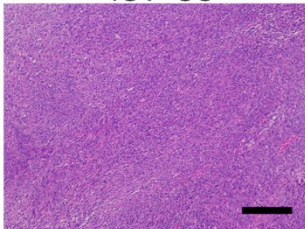

IGVs+Laser

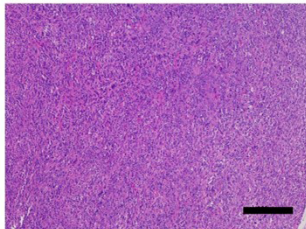

IGVs+US+Laser

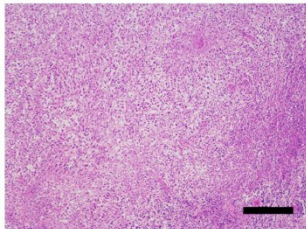

Supplement: S8 Fig — H&E staining of tumors after different treatment. Scale bar = 200 μm. (PDF) [file pbio.3003786.s010.pdf]
