# Supplementary material for: Acoustic delivery of indocyanine green via biosynthetic gas vesicles for tumor photothermal therapy
Source: PLoS Biol. 2026 May 13;24(5):e3003786. doi: 10.1371/journal.pbio.3003786 (PMC13170872; doi:10.1371/journal.pbio.3003786)
Supplement: Attachment — Submitted filename: Response to Reviewers_0226.docx [file pbio.3003786.s013.docx]

**RESPONSES TO REVIEWS’ COMMENTS**

Reviewer #1: The authors present a biosynthetic gas vesicle (GV)-based delivery system for indocyanine green (ICG) that addresses several intrinsic limitations of the only FDA-approved photothermal agent, including poor aqueous stability, a short in vivo half-life, and insufficient tumor-targeted accumulation. In this system, ICG is covalently conjugated to the surface of GVs via amide bonds, yielding a nanoscale probe with an average size of approximately 264 nm. The resulting ICG-GVs enable multimodal imaging, including ultrasound, near-infrared fluorescence, and photoacoustic imaging, allowing real-time visualization of ICG delivery to tumors. Importantly, ultrasound-induced cavitation enhances cellular uptake of ICG, and when combined with 808 nm laser irradiation, the platform achieves an in vitro tumor cell killing rate exceeding 90%. In vivo, tumor temperatures reach up to 64.2 °C, leading to effective tumor regression and prolonged survival in tumor-bearing mice. The system exhibits favorable biosafety, with no significant organ toxicity, highlighting its potential as a controllable, precise, and clinically translatable strategy for ICG-mediated photothermal therapy.

Despite these promising results, several issues should be addressed to further strengthen the manuscript and enhance its translational value.

**Comment 1:** The authors do not clearly compare ICG-GVs with clinically used ultrasound microbubbles in terms of ICG loading efficiency, in vivo circulation half-life, and tumor-targeting performance. Such comparisons are essential to demonstrate the unique advantages of GVs.

**Response 1:** We thank you for this important suggestion. We fully agree that the direct comparison between our ICG-GVs platform and clinically widely used ultrasound microbubbles is critical for highlighting the innovation and advantages of our study. However, after an in-depth analysis of the structural characteristics of currently available commercial microbubbles, we have identified a fundamental technical obstacle within the framework of our current study.

(1) Commercial microbubbles lack surface functional groups for stable covalent conjugation of ICG.

The most widely used clinical ultrasound microbubbles, such as SonoVue® (phospholipid-shelled sulfur hexafluoride microbubbles) and Sonazoid® (hydrogenated egg phosphatidylserine-shelled perfluorobutane microbubbles), are primarily composed of phospholipid shells. The hydrophilic head groups of these phospholipids typically consist of moieties such as phosphocholine, which lack reactive functional groups (e.g., amino, carboxyl, or thiol groups) available for chemical conjugation. This structural limitation precludes the application of the same covalent conjugation strategy (amide bond formation) that we successfully employed for ICG-GVs. If ICG were loaded onto these commercial microbubbles via physical adsorption or non-covalent encapsulation, the ICG would dissociate rapidly upon systemic circulation. Such unstable loading would not accurately reflect the true potential of "microbubble-mediated delivery" and could potentially yield misleading comparative data regarding in vivo stability, circulation half-life, and targeting performance.

(2) Covalent conjugation represents the core advantage of the GV platform over conventional microbubbles

A key rationale for selecting biosynthetic GVs as our delivery vehicle is the natural presence of surface-accessible amino groups, enabling stable covalent conjugation of ICG via amide bonds. This stable linkage confers excellent structural and functional stability to ICG-GVs, which is fundamental to realizing the "visibly acoustic delivery" concept proposed in our study. In essence, the inability of conventional commercial microbubbles to undergo covalent modification is precisely where the GV platform demonstrates its core innovation and inherent advantage.

(3) We have indirectly demonstrated the delivery advantages of the GV platform through comparisons with appropriate control groups

Although a direct chemically equivalent comparison with commercial microbubbles is not feasible, we have incorporated extensive experimental data in the revised manuscript to demonstrate the advantages of the GV platform through rigorous comparisons with ICG-GVs without ultrasound triggering:

Through quantitative analysis of intratumoral ICG accumulation (Fig. 4F-H) and real-time monitoring of in vivo photothermal effects (Fig. 5E-F), we unequivocally demonstrated that ultrasound-triggered GV cavitation significantly enhanced tumor-targeted delivery and therapeutic efficacy. These data robustly support the unique value of the GV platform in ultrasound-mediated active delivery.

In conclusion, due to the lack of surface functional groups amenable to stable covalent ICG conjugation on commercial microbubbles (e.g., SonoVue®, Sonazoid®), the direct comparison within the technical framework of our study is scientifically unfeasible. Through rigorous comparisons with non-triggered ICG-GVs, we have robustly demonstrated the unique advantages of the GV platform in ultrasound-enhanced tumor-targeted delivery. We have added a discussion of this technical limitation in the revised manuscript to provide readers with a clearer scientific context.

We thank you once again for this valuable input, which has allowed us to more clearly articulate the technical innovation and scientific significance of our study.

**Comment 2:** Key parameters relevant to clinical translation, including large-scale production feasibility, batch-to-batch consistency, and storage stability, are not discussed.

**Response 2:** We thank you for highlighting the importance of parameters relevant to clinical translation. We fully agree that large-scale production feasibility, batch-to-batch consistency, and storage stability are critical indicators for evaluating the translational potential of novel nanoplatforms. We would like to address these points as follows:

(1) Gas vesicles (GVs) can be produced via microbial fermentation, offering the feasibility for large-scale production.

The biosynthetic GVs used in this study are derived from fermentative culture of *Halobacterium NRC-1*. As a biologically derived nanomaterial, GVs can be obtained through microbial fermentation in a relatively controlled manner, providing a favorable foundation for clinical translation. Our team has currently systematically optimized the fermentation parameters and realized the 100L scale-up fermentation, just showed as the following picture.

Figure Q&A 1. Scale-up fermentation of GVs-producing *Halobacterium NRC-1*.

(2) The conjugation reaction is mild and straightforward, and free ICG is readily removable.

The conjugation of ICG to GVs employs a classic amidation reaction, using the naturally occurring amino groups on the GV surface, which can be completed under mild conditions (4°C, 3 h). Unconjugated free ICG can be efficiently removed through repeated centrifugation washing or ultrafiltration, a simple and reproducible procedure. This conjugation-purification workflow requires no specialized equipment and exhibits good potential for scale-up.

(3) Based on the controllability of the reaction mechanism, batch-to-batch consistency is theoretically achievable.

Based on the two points above—namely, the relatively stable surface amino group density of GVs (stemming from the controllability of biosynthesis) and the simple and reliable conjugation-purification process—we believe that batch-to-batch consistency of ICG-GVs is theoretically achievable. We will systematically collect multi-batch data in future work to further validate this inference experimentally. Regarding storage stability, we have provided preliminary data demonstrating the stability of ICG-GVs in PBS over 7 days in the Supplementary Material (Fig. S2), confirming its good storage stability; longer-term stability studies will be conducted in future work. We have added a brief discussion of these translational parameters in the revised manuscript to provide readers with a more comprehensive scientific context.

Figure S2. Long-term stability of ICG-GVs. The stability of ICG-GVs was monitored in PBS over 7 days. Data are presented as mean ± SD (n = 3 per group).

**Comment 3:** Although ICG is covalently coupled via amide bonds, the manuscript does not report quantitative values for ICG loading capacity or encapsulation efficiency.

**Response 3:** We thank the reviewer for this observation. According to your suggestion, we have performed quantitative measurements of ICG loading capacity. The experimental results demonstrate that ICG loading efficiency was 58%, indicating that the amide bond-mediated covalent conjugation strategy achieves efficient ICG loading with good batch-to-batch reproducibility.

**Comment 4:** Only a single ultrasound condition (−3 dB power, 10 s interval) is used throughout the study, without justification or optimization.

**Response 4:** We thank you for raising this important point regarding the ultrasound parameters. The ultrasound conditions used in this study (−3 dB power, 10 s interval) were not arbitrarily selected, but rather based on a series of previous studies from our group on gas vesicle (GV) ultrasound imaging.

(1) Literature basis for parameter selection

Our group has systematically investigated the ultrasound imaging performance of GVs in multiple published studies, validating the efficacy of comparable ultrasound parameters. For example:

In a study published in Pharmaceutics (2022), our team used clinical diagnostic ultrasound equipment and confirmed that GVs isolated from *Halobacterium NRC-1* could produce stable and strong ultrasound contrast signals in tumors under optimized parameters (PMID: 35745771). This study provided important references for selecting ultrasound parameters for GV imaging.

In a recent study published in Ultrasound in Medicine and Biology (2025), we performed ultrasound molecular imaging of GVs using clinical diagnostic equipment (Mindray Resona 9T) with parameters comparable to those used in this study (PMID: 40517079).

Furthermore, in multiple targeted imaging studies published in Small (2023), Biomaterials (2023), and other journals, we employed routine settings of clinical diagnostic ultrasound equipment (including the −3 dB power range) for GVs ultrasound imaging detection (PMID: 36866487, 36566551).

(2) Clinical relevance of the −3 dB parameter

The −3 dB power setting corresponds to routine operating conditions within the safe mechanical index (MI) range for clinical diagnostic ultrasound. According to the American Institute of Ultrasound in Medicine (AIUM) safety guidelines, the mechanical index for diagnostic ultrasound is typically maintained below 1.9, and the −3 dB setting ensures acoustic output remains within safety limits. The parameters we used (−3 dB power, 10 s interval) are consistent with those validated in the team's previous studies and can induce stable GV cavitation effects without causing tissue damage.

(3) Sufficiency of this parameter for the current study

Within the technical framework of this study, this single ultrasound parameter has proven sufficient to demonstrate the core scientific question: ultrasound-triggered GV cavitation significantly enhances tumor delivery of ICG (Fig. 4F-H) and photothermal therapeutic efficacy (Fig. 5E-F, Fig. 6). We agree with the reviewer that systematic optimization of ultrasound parameters (e.g., different mechanical indices, pulse lengths, duty cycles) would further improve delivery efficiency, and this represents an important direction for our future research.

We have added the literature basis for parameter selection in the revised Materials and Methods section and included a future perspective on ultrasound parameter optimization in the Discussion section. We thank the reviewer again for this valuable suggestion.

**Comment 5:** The scale bar in the TEM image (Figure 2C) is listed as 200 nm but is not clearly labeled. In addition, the grouping information in the fluorescence images (Figures 4B and 4C) is ambiguous, particularly regarding the presence or absence of ultrasound irradiation.

**Response 5:** We thank you for their careful observation. We have re-examined the relevant figures and revised the labeling as follows:

Figure 2C: The scale bar in the TEM image is clearly indicated as "200 nm" and presented as a white line within the image. To enhance readability, we have bolded the scale bar line and adjusted the font contrast in the revised version to ensure its clarity.

Figure 2(C). TEM micrographs showing the morphology of GVs and ICG-GVs. Scale bar = 200 nm.

Figure 4B and 4C: The grouping information is clearly labeled. In Figure 4B, the group labels are positioned on the left side of the images (from top to bottom: ICG-GVs, ICG-GVs+US). In Figure 4C, the group labels are placed either above or below each individual sub-image ("+US" explicitly indicate the presence of ultrasound irradiation). All labels are clearly legible in both print and electronic formats.

Figure 4. Ultrasound irradiation enhanced ICG delivery into tumor cells. (A) Schematic illustration of cavitation enhances the delivery of ICG into the tumor cells in vitro. (B) The fluorescence images of MB49 cells incubated with ICG-GVs either with acoustic burst or without acoustic burst. Scale bar = 20 μm. (C) The fluorescence images and (D) quantification of MB49 cells after photothermal treatment. Viable cells were stained green with calcein-AM, and dead/later apoptosis cells were stained red with PI. Scale bar = 200 μm.

**Comment 6:** The tumor growth curve in Figure 6C displays only mean values without individual data points, obscuring data variability. Overlaying individual measurements would improve transparency.

**Response 6:** We thank the reviewer for this valuable suggestion. We have replotted the tumor growth curves as requested. In the revised Figure 6C, individual data points are now overlaid on the mean curves. The modified figure clearly displays the individual tumor growth trajectories of each mouse (n=8 per group) as scattered points, while retaining the mean ± SEM line connections to illustrate overall trends. This revision significantly enhances both the transparency and informativeness of the data presentation.

Figure 6(C). The tumor growth curves of mice received different treatments. Data are mean ± SD (n=8 per group). Two-way ANOVA. ^**^P<0.01 vs. control.

**Comment 7:** The "Key Findings" sections in Tables 1 and 2 are overly verbose and partially redundant with the main text.

**Response 7:** We thank you for their careful reading. We would like to clarify that the manuscript submitted in this revision does not contain any tables. After thoroughly checking the current submission, we confirm that there are no Table 1 or Table 2 included in the main text—only the main text, figures, and figure legends are present.

**Comment 8:** The treatment scheme in Figure 6A does not specify key ultrasound (power, duration, interval) and laser (wavelength, power density, irradiation time) parameters. Likewise, the quantitative imaging data in Figure 3D-F do not clearly define the measured indicators (for example, fluorescence intensity, signal-to-noise ratio).

**Response 8:** We thank you for this careful observation.

About the Figure 6A, following the reviewer's suggestion, we have modified Figure 6A in the revised manuscript to directly annotate these key parameters on the schematic, with text boxes added next to the ultrasound probe ("US: 3.0–11.0 MHz, -3 dB, every 10 s") and laser icon ("Laser: 808 nm, 0.75 W, 20 min"), along with complete parameter descriptions included at the end of the figure legend to enhance clarity and facilitate readers' understanding of the experimental design. Also, the ultrasound and laser parameters were already described in detail in the Materials and Methods section (Section 2.6), including ultrasound parameters (3.0–11.0 MHz linear array transducer, -3 dB power, bursts every 10 seconds until ICG-GVs signal disappeared) and laser parameters (808 nm diode laser, 0.75 W power, 20 minutes irradiation at 4 hours post-injection).

Figure 6(A). Schematics of acoustic ICG delivery tumor photothermal therapeutic regimen.

About the quantitative imaging data in Figure 3D-F, we have revised Figure 3D-F to clearly define the measured indicators: the labels in Figures 3D, 3E, and 3F have been modified to "CEUS signal intensity ", "PA signal intensity", and "NIRF signal intensity ", respectively. Additionally, we have supplemented the figure legends to explicitly state that Figure 3D corresponds to quantitative analysis of CEUS imaging (Figure 3A), Figure 3E corresponds to PA imaging (Figure 3B), and Figure 3F corresponds to NIRF imaging (Figure 3C). The Results section (3.2) has also been enhanced with explicit descriptions of Figures 3E and 3F, detailing the quantitative relationship between ICG-GVs concentration and photoacoustic/fluorescence signal intensities. These modifications ensure that all quantitative indicators are clearly defined for accurate interpretation of the experimental data.

Figure 3(D) Quantitative analysis of CEUS signal intensity corresponding to (A). Data are mean ± SD (n=3 per concentration). (E) Quantitative analysis of PA signal intensity corresponding to (B). Data are mean ± SD (n=3 per concentration). (F) Quantitative analysis of NIRF signal intensity corresponding to (C). Data are mean ± SD (n=3 per concentration).

**Comment 9:** Several abbreviations, including CEUS, PA, and PTT, are not defined at their first appearance in the figure captions, which does not conform to standard academic practice.

**Response 9:** We thank you for this careful observation. We have thoroughly reviewed the entire manuscript and corrected all abbreviations at their first appearance in the figure captions. We have ensured that all abbreviations in the figure captions are properly defined at their first appearance throughout the manuscript, in accordance with standard academic publishing practices.

**Comment 10:** Most figure captions do not report sample sizes (n values) or statistical analysis methods, relying instead on descriptions in the main text.

**Response 10:** We thank you for this careful observation. We have revised all figure legends throughout the manuscript and supplemented sample sizes (n values) for all quantitative data figures, uniformly formatted as "Data are presented as mean ± SD (n=X per group)" or "Data are presented as mean ± SEM (n=X per group)". For all figures involving statistical analysis, the figure legends now explicitly state the statistical methods used (e.g., unpaired t-test, one-way ANOVA, two-way ANOVA, log-rank test) and the significance levels.

**Comment 11:** The reference format needs to be unified, and some important references are missing, such as "Ultrasound-controlled drug release and drug activation for cancer therapy" and "Recent advances in indocyanine green-based probes for second near-infrared fluorescence imaging and therapy." Ensure that the reference format is consistent and check for any missing important references.

**Response 11:** We have unified the reference format according to the journal style and added some important references including the two references stated above.

Reviewer #2: In this manuscript, Zhang et al. developed biosynthetic gas vesicles coated with ICG, which are designed to enhance and visualize the delivery of ICG and improve its anti-tumor efficacy in PTT. While the study presents an interesting approach, several serious concerns must be raised regarding the unclear scientific premise and significant weaknesses in the animal experiments described.

Major critics:

**Comment 1:** The manuscript claims that acoustic cavitation enhances ICG delivery into tumor cells, while the discussion of its underlying mechanism is inadequate. Please discuss the potential mechanism.

**Response 1:** We thank you for this suggestion. We have added some discussion about the potential mechanisms of acoustic cavitation-enhanced ICG delivery in the revised manuscript. Please find them marked in red in the revised manuscript.

**Comment 2:** Regarding the pharmacokinetic evidence, the manuscript claims improved delivery stability for ICG-GVs, but a critical piece of evidence is missing: a direct comparison of the in vivo half-life between ICG-GVs and free ICG.

**Response 2:** We thank you for this suggestion. We have performed the direct comparison of the in vivo half-life between ICG-GVs and free ICG. Briefly, healthy mice were intravenously injected with equal doses of free ICG or ICG-GVs (based on the ICG amount), and blood samples were collected at 1, 5, 10, 30 minutes and 1, 2, 4, 8, 12, 24 hours post-injection for plasma ICG concentration measurement. The results showed that plasma concentration in the free ICG group declined rapidly, falling below the detection limit within 4 minutes, with a half-life of approximately 3.2 ± 0.5 minutes. In contrast, ICG-GVs reached half of the initial concentration at 20 minutes post-injection, with a half-life extended to 22.5 ± 3.1 minutes. These data directly demonstrated that ICG-GVs significantly prolong the blood circulation time of ICG and improve its in vivo delivery stability. The relevant results have been incorporated into the revised Figure S4.

Figure S4. Pharmacokinetic analysis of ICG-GVs and free ICG in healthy mice. Plasma concentration-time profiles of ICG after intravenous injection of free ICG or ICG-GVs (ICG dose: 2 mg/kg) in healthy C57BL/6 mice. Blood samples were collected at indicated time points and ICG concentration was measured by fluorescence spectrophotometry. Data are presented as mean ± SD (n = 3 per group).

**Comment 3:** It is necessary to update both the manuscript and the figures to clearly specify the NIR and ultrasound parameters and the injection regimen of ICG-GVs in vivo anti-tumor therapy.

**Response 3:** We thank you for this suggestion. In response, we have now clearly specified all key parameters for the in vivo anti-tumor therapy in the revised manuscript:

(1) Injection regimen: Mice were intravenously injected via the tail vein with ICG-GVs (OD_500_=3.0, corresponding to an ICG concentration of 40 μg/mL) at a single dose volume of 100 μL.

(2) Ultrasound parameters: Ultrasound was performed using a Mindray Resona 7 system equipped with an L11-3U linear array transducer (3.0–11.0 MHz). When CEUS imaging confirmed complete tumor perfusion of ICG-GVs, burst acoustic irradiation was applied at -3 dB power (mechanical index MI < 1.9, within diagnostic ultrasound safety limits) with pulses every 10 seconds until the ICG-GVs signal disappeared.

(3) Laser parameters: At 4 hours post-injection, tumors were irradiated with an 808 nm diode laser at 0.75 W for 20 minutes, and tumor surface temperature was monitored in real-time using an FTIR thermal camera (FLIR E4).

These parameters have been described in detail in the Materials and Methods section 2.6, directly annotated on the treatment schematic in Figure 6A ("US: 3.0–11.0 MHz, -3 dB, every 10 s"; "Laser: 808 nm, 0.75 W, 20 min"), and fully explained in the corresponding figure legend.

**Comment 4:** The clinical relevance of this study hinges on the safety of intravenous ICG administration. It is essential to include in vivo safety assessments, such as serum biochemistry, complete blood counts, and histopathological analysis of major organs, to rule out any adverse toxic effects.

**Response 4:** We thank you for highlighting the importance of safety assessment for clinical translation. In response, we have performed the comprehensive in vivo safety evaluation, including serum biochemistry, complete blood counts, and histopathological analysis of major organs.

Briefly, healthy C57BL/6 mice were treated with either PBS (control group) or ICG-GVs+US+Laser (same conditions as the therapeutic study). Blood samples and major organs (heart, liver, spleen, lungs, kidneys) were collected at days 1 and 7 post-treatment for analysis.

Complete blood counts (Fig. S5): Hematological parameters including white blood cell count, red blood cell count, hemoglobin, and platelet count showed no significant differences between control and treatment groups, indicating no treatment-induced hematotoxicity.

Liver and kidney function markers (Fig. S6): Serum levels of alanine aminotransferase (ALT), aspartate aminotransferase (AST), blood urea nitrogen (BUN), and creatinine (CREA) were comparable between groups, suggesting no hepatic or renal impairment.

Histopathological analysis (Fig. S7): H&E staining of heart, liver, spleen, lungs, and kidneys revealed normal tissue architecture in the treatment group, with no obvious inflammatory infiltration, cellular necrosis, or fibrosis, consistent with the control group.

These data collectively demonstrate that ICG-GVs combined with ultrasound and laser irradiation exhibit a favorable biosafety profile in vivo, with no detectable toxic effects. The relevant results have been incorporated into the revised Supplementary Figures S5-S7. We thank the reviewer again for this valuable suggestion, which has substantially strengthened the translational relevance of our study.

Figure S5. ICG-GVs+US+Laser treatment had no hematological toxicity. Hematology profiles, (A) Hematocrit (HCT), (B) white blood cell count (WBC), (C) platelet count (PLT), (D) hemoglobin (HGB), (E) red blood cell count (RBC), (F) mean corpuscular hemoglobin (MCH), (G) mean corpuscular volume (MCV), and (H) mean corpuscular hemoglobin concentration (MCHC), show no significant differences between the control and the ICG-GVs + US + Laser treatment groups.

Figure S6. Serum levels of alanine aminotransferase (ALT), aspartate aminotransferase (AST), blood urea nitrogen (BUN), and creatinine (CREA).

**Figure S7. Absence of organ damage.** H&E staining of major organs (heart, liver, spleen, lung, kidney) in control and ICG-GVs group. Scale bar =200 μm.

**Comment 5:** The biodistribution data shows predominant accumulation of ICG-GVs in the liver following intravenous administration. To more rigorously evaluate the translational potential for treating deep-seated tumors, it is strongly recommended to include an orthotopic liver cancer model in the study.

**Response 5:** We sincerely thank you for your thorough review and valuable suggestions. We fully agree its significance to introduce an orthotopic liver cancer model for validating tumor targeting is critically important for enhancing the clinical translational value of our study. However, after a comprehensive assessment of current technical constraints and the core objectives of our research, we are sorry to tell you that we face some technical obstacles to establish this tumor model. We wish we can provide these data in our future paper. Although the biodistribution data shows predominant accumulation of ICG-GVs in the liver following intravenous administration, the relative delivery amount of ICG into tumor have been significantly improved. It needs to point out that the core innovation of our study lies in developing a "visibly acoustic delivery" platform based on biosynthetic gas vesicles (GVs), aimed at addressing the key bottleneck of tumor-targeted delivery of free indocyanine green (ICG).

Minor issues:

**Comment 6:** The color scale bars in the figures should be revised. Please replace the non-quantitative labels "High" and "Low" with specific numerical values. Additionally, scale bars are missing in several figures (Fig. 2B, Fig. 3H, Fig. 3I, Fig. 4F, Fig. 4G, Fig. 5A).

**Response 6:** We thank you for this valuable suggestion and have thoroughly revised all figures in the manuscript accordingly. All pseudo-colored images previously labeled with qualitative "High/Low" indicators have been changed into specific numerical signal intensity ranges. Additionally, physical scale bars have been added to all images previously missed, enabling readers to accurately interpret the actual dimensions of the displayed images.

**Comment 7:** Some of the presented data lack appropriate statistical analysis. Please add the necessary statistical analysis and specify the methods used (e.g., unpaired t-test, one-way ANOVA).

**Response 7:** We thank you for this careful observation. In response, we have revised all figure legends throughout the manuscript to include sample sizes (n values) for all quantitative data, uniformly formatted as "Data are presented as mean ± SD (n = X per group)" or "Data are presented as mean ± SEM (n = X per group)". Additionally, for all figures involving statistical analysis, the figure legends now explicitly state the statistical methods used (e.g., unpaired t-test, one-way ANOVA, two-way ANOVA, log-rank test) and the corresponding significance levels.
